# Variation Quality and Kinetic Parameter of Commercial *n*-3 PUFA-Rich Oil during Oxidation via Rancimat

**DOI:** 10.3390/md15040097

**Published:** 2017-03-28

**Authors:** Kai-Min Yang, Po-Yuan Chiang

**Affiliations:** Department of Food Science and Biotechnology, National Chung Hsing University, 250 Kuokuang Road, Taichung 40227, Taiwan; a9241128@gamil.com

**Keywords:** *n*-3 PUFA, oxidative stability index, Rancimat test, kinetic parameter

## Abstract

Different biological sources of *n*-3 polyunsaturated fatty acids (*n*-3 PUFA) in mainstream commercial products include algae and fish. Lipid oxidation in *n*-3 PUFA-rich oil is the most important cause of its deterioration. We investigated the kinetic parameters of *n*-3 PUFA-rich oil during oxidation via Rancimat (at a temperature range of 70~100 °C). This was done on the basis of the Arrhenius equation, which indicates that the activation energies (*E*a) for oxidative stability are 82.84–96.98 KJ/mol. The chemical substrates of different oxidative levels resulting from oxidation via Rancimat at 80 °C were evaluated. At the initiation of oxidation, the tocopherols in the oil degraded very quickly, resulting in diminished protection against further oxidation. Then, the degradation of the fatty acids with *n*-3 PUFA-rich oil was evident because of decreased levels of PUFA along with increased levels of saturated fatty acids (SFA). The quality deterioration from *n*-3 PUFA-rich oil at the various oxidative levels was analyzed chemometrically. The anisidine value (p-AV, r: 0.92) and total oxidation value (TOTOX, r: 0.91) exhibited a good linear relationship in a principal component analysis (PCA), while oxidative change and a significant quality change to the induction period (IP) were detected through an agglomerative hierarchical cluster (AHC) analysis.

## 1. Introduction

Fish oil accounts for less than 1% of all the global edible oil produced, but it is the main source of *n*-3 PUFA, specifically, eicosapentaenoic acid (EPA) and docosahexaenoic acid (DHA) [1]. These oils are becoming increasingly popular with consumers in view of the clear evidence of the health benefits of *n*-3 PUFA for individuals with cardiovascular conditions, including their beneficial role as antithrombotic, anti-inflammatory, and hypolipidemic fatty acids [2,3]. The *n*-6/*n*-3 ratio in a person’s dietary intake is also an important consideration due to its influences on cardiovascular health and inflammation, with high intake of *n*-6 PUFA potentially attenuating the known profitable effects of *n*-3 PUFA. Due to the nutritional changes described above in the Western diet, the *n-*6/*n*-3 ratio has now increased to falls between 10 and 20. The dietary recommendations regarding *n*-3 PUFA intake are, thus, of increasing importance [4].

In Europe, the recommended daily intake of EPA and DHA is 450 mg per day, that is, around 3 g per week, while the WHO/FAO recommends daily consumption of 250 mg (for primary prevention) to 2 g (for secondary prevention) of EPA and DHA to prevent cardiovascular conditions [5]. In contrast, the FDA and American Dietetic Association suggest a minimum intake of close to 500 mg/day to prevent coronary health diseases [6]. In recent years, consumers have identified *n*-3 PUFA supplements as options for reducing the probability of illness and avoiding expensive medical bills, so that the rate of sales growth for such supplements is currently around 15% per annum. The calculated market value of packaged products containing *n*-3 PUFA, which primarily consist of infant formula, has been estimated to reach $34.7 billion by 2016, and to grow at a CAGR of 9.1% from 2015 to 2022 [7].

Worldwide fish stocks peaked some years ago, but in recent years there has been a shortage in the supply of fish oils. In the future, as the global population continues to grow, in turn increasing the need for products allowing consumers to meet the suggested intake of EPA and DHA, sustainable sources of *n*-3 PUFA-containing products will be needed to meet the growing demand [8,9]. With that in mind, the use of other forms of marine life, including Antarctic krill and algae, to provide *n*-3 PUFA continues to be developed, with these sources already having been commercialized. These sources may provide some high value and highly concentrated products for human consumption, including contaminant-free products with good sensory qualities that are also safe and environmentally friendly. In particular, the fermentation of algae can be used to shorten the growth process of algae and produce highly concentrated oil [10,11].

The drawback of using *n*-3 PUFA-rich oils for functional foods is that they are readily oxidized in the presence of oxygen, heat, light, and metal ions, and the secondary products of lipid oxidation can impair the sensory qualities and acceptability of products among consumers [12]. In addition, previous studies involving animal research have confirmed that oxidative products contain genotoxic and cytotoxic compounds [13]. Moreover, these oxidative compounds, when present in diets, have been considered as the possible causative agents of several diseases, such as chronic inflammation, neurodegenerative diseases, atherogenesis, diabetes, and certain types of cancer. Among these oxidative products, the oxygenated aldehydes are the most broadly studied, and their adsorption capacity and functional group profiles are most closely related to toxicity [13,14].

The Global Organization for EPA and DHA (GOED) voluntary monograph is the quality standard for EPA- and DHA-rich oils, and is used to help ensure that consumers have access to high-quality products; it is applicable to the EPA and DHA fatty acids obtained from fish, plant, or microbial sources [15]. Numerous analytical methods of lipid oxidation are used to measure food quality. However, there is no common and standard method for detecting all oxidative variations in multiple food systems. Therefore, it is necessary to select a proper and adequate method for the analysis of any fatty acid composition and its substrates.

Lipid oxidation occurs very slowly at room temperature and, hence, accelerated methods should be applied in order to estimate the oxidative stability of a product or the induction time of the autoxidation reaction in a more rapid manner, especially as the temperature and the rate of said reaction are exponentially related [16]. Well-established accelerated aging testing methods include the active oxygen method, Schaal Oven test, and Rancimat test. For the determination of IP, which is the time needed for oil deterioration to commence, the Rancimat test observes the changes to the conductivity of samples while the other two methods look at peroxide value (POV). So the Rancimat test is easy to use and has good reproducibility [17]. The Rancimat method has been widely used to evaluate the shelf lives of various products, including the kinetic parameters of antioxidants in oil samples, as well as the inhibition of lipid peroxidation in such antioxidants [16,17,18].

The Rancimat test promotes the oxidation process by exposing oil samples to a high temperature or temperatures and a sufficient amount of oxygen. In the current study, we collected oils with different oxidation levels during the induction period. This study discusses the evolution of substrate changes and the formation of primary and secondary oxidation products, which were characterized in terms of oxidative stability through the use of agglomerative hierarchical cluster AHC analysis and PCA. These parameters were used to evaluate the oxidative properties of *n*-3 PUFA-rich oil, and the resulting information can further be used to control the stability and shelf lives of PUFA-containing products.

## 2. Results and Discussion

### 2.1. Kinetic Analysis

**Figure 1:**
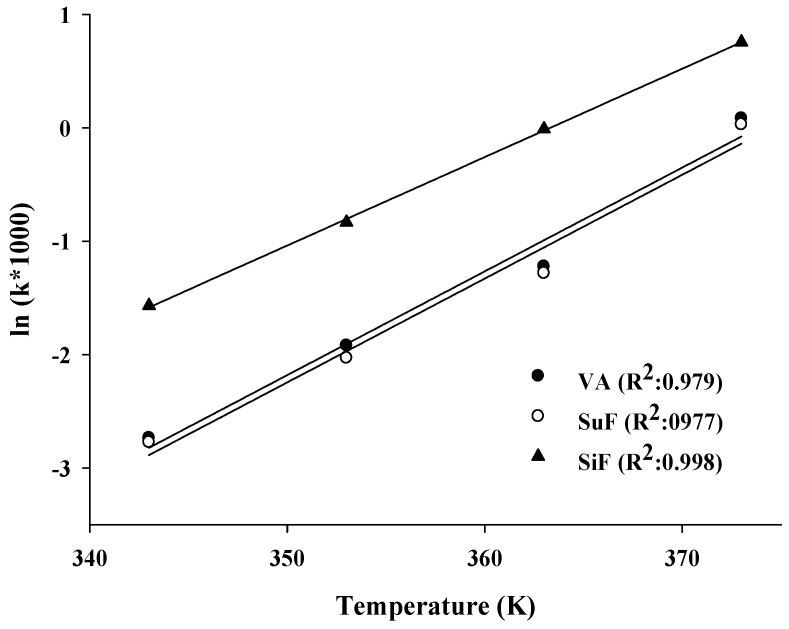
Semi-logarithmic relationship between k and temperature values for lipid oxidation of the *n*-3 PUFA-rich oils.

Given the uncertainty regarding the best temperature conditions for accelerated methods, the oxidative stability of *n*-3 PUFA-rich oils was studied using the Rancimat test with temperatures ranging from 70 °C to 100 °C. The IP for the lipid oxidation of *n*-3 PUFA-rich oils at different temperatures are presented in Figure 1. For use of the Rancimat test at temperatures of 70 °C, 80 °C, 90 °C, and 100 °C, the induction times were 15.4, 6.8, 3.4, and 0.92 h, respectively, for the VA; 15.9, 7.6, 3.6, and 0.97 h, respectively, for the SuF; and 4.8, 2.3, 1.01, and 0.47 h, respectively, for the SiF. The temperature used affects the degree of oxygen solubility in a given oil sample, with the oxygen solubility decreasing by almost 25% for each 10 °C rise in temperature [19]. Generally, the induction time was halved with each 10 °C increase in temperature. Previous research has shown that the PIs (at 50~80 °C) of fish oils without antioxidants ranged from 24.3~0.6 h, and that the IP of the same oils ranged from 52.3~2.4 h when 400 ppm of α-tocopherol were added [20].

Many studies have shown that the Rancimat test can be used to determine and evaluate the kinetic parameters of oils. The determination of such kinetic parameters is valuable for the purpose of distinguishing the origins of various oils, for characterizing the differences or similarities in the oils, and for predicting the oxidative stability of oils under various storage conditions [17,19]. In this study, there was semi-logarithmic relationship with Equation (1) for all the oil samples, including a linear dependency with good correlation of determination, with *R*^2^ being 0.979 for the VA, 0.977 for the SuF, and 0.998 for the SiF (Figure 1).

The *E*a value is of interest for the properties of oils, which demonstrates the delay of the initial oxidation reaction due to the bond scission that takes place to form primary oxidation products [21]. Table 1 shows *E*a values of the assayed oils were 96.98 kJ/mol for the VA, 96.97 kJ/mol for the SuF, and 82.84 kJ/mol for the SiF (Table 1). The *E*a is influenced by unsaturated number of oil samples, as the *E*a seems to be lower for oils with higher PUFA levels. According to the reference, the *E*a values of DHA and EPA ethyl esters (of 95–97% purity), which were in the range of 52.1~62.4 kJ/mol [22]. However, this is contradicted by the example of the VA assayed in this study. Specifically, while the PUFA content of the VA was higher than those of the other tested oils, because the process of molecular distillation can increase the IP of VA, it had a higher *E*a value than the other oils [23].

### 2.2. Monitoring Substrate Variants

As shown in Table 2, we identified eight types of fatty acids as presented under the VA column, 12 types of fatty acids as presented under the SuF column, and 14 types of fatty acids as presented under the SiF column. We observed that the constituents of 100 g of VA consisted of 34.4 g of SFA, 34.5 g of MUFA, and 30.3 g of PUFA, while 100 g of SuF contained 44.5 g of SFA, 20.2 g of MUFA, and 17.6 g of PUFA. One hundred grams of SiF consisted of 43.9 g of SFA, 28.0 g of MUFA, and 24.0 g of PUFA. The concentration (g/100 g) of *n*-3 PUFA was 26.8 for VA, 16.4 for SuF, and 16.3 for SiF. The literature on this topic indicated that DHA could be efficiently synthesized in microalgae via an anaerobic pathway involving polyketide synthases [11].

**Figure 2:**
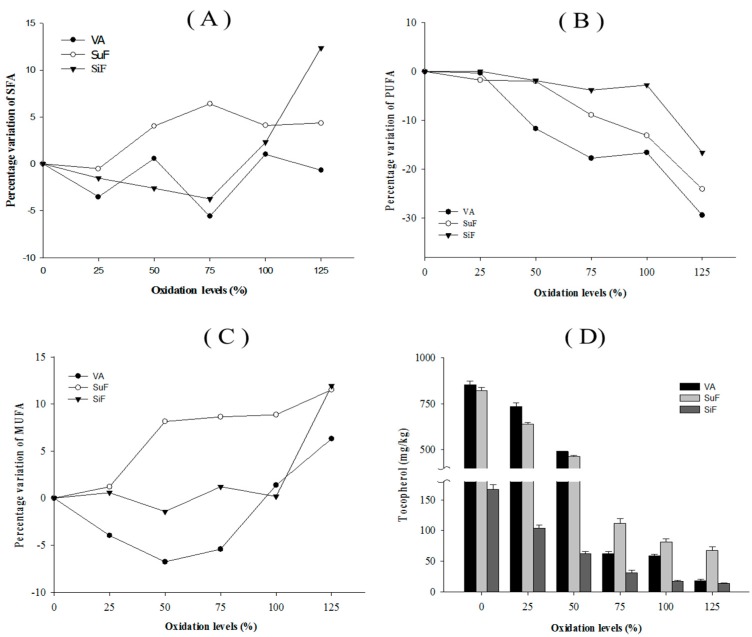
Percentage variations (g/100 g oil) of the (**A**) SFA, (**B**) MUFA, (**C**) PUFAs, and (**D**) total tocopherol measured in the *n*-3 PUFA-rich oils.

The combination of fatty acids in edible oil is the most important factor in determining the oil’s oxidation stability. Processing sophistication and antioxidants can improve the oxidation stability of commercial products. The levels of fatty acids change at 80 °C under the Rancimat test (Figure 2), with the difference in the PUFA amounts of the VA, SuF, and SiF blends being −16.60%, −13.11%, and −2.76% at the 100% oxidation level, and −29.43%, −24.08%, and −16.61% at the 125% oxidation level, mainly as a result of DHA degradation. The difference in the SFA amounts of the VA, SuF, and SiF blends were 1.01%, 4.11%, and 2.29% at the 100% oxidation level, and −0.67%, 4.34%, and 12.36% at the 125% oxidation level. It is known that the thermal treatment of oils and fats generates hydroperoxide breakdown of any fatty acids with a chain shorter than ten carbon atoms, such that such breakdown can be considered a chemical indicator of the fat degradation grade [24]. This study was similar to such results, as SFA formation appeared to be correlated with PUFA loss, and with monounsaturated fatty acid (MUFA) increases and decreases.

Tocopherols are the most abundant antioxidants in *n*-3 PUFA concentrates, because of their capacity to inhibit hydroperoxides and C-3 aldehydes [25]. In commercial products, tocopherol can be used independently or in combination with other compounds, such as ascorbic acid palmitate, lecithin, and catechin, and has shown significantly in fish oil stabilization. On the other hand, the structure conformation also affects the physical property of tocopherol, as γ- and δ-tocopherol have thermal resistance. Hydroperoxyl radical-scavenging activity occurs in the order of α- > β- > γ- > δ-tocopherol [26]. As shown in Figure 2, half of the total tocopherols of the tested *n*-3 PUFA-rich oils was lost at the 50% oxidation level under the Rancimat test, while 90% of the total was lost at the 100% oxidation level under the Rancimat test. The fact that the tocopherol content dropped very quickly under the Rancimat test conditions could be due mainly to the very high susceptibility of this molecule to oxidation to tocopherol quinones at high temperatures, which diminishes the protection of unsaturated fatty acids against oxidation [27].

### 2.3. Monitoring Oxidation Products

Lipid oxidation products have negative impacts on the flavor and odor of sensory parameters, which can, in turn, have harmful effects on human health. The progress of lipid oxidation can be evaluated by the monitoring of a diverse series of primary, secondary, and tertiary oxidation products over time. The quality standards of GOED require specific levels of acid value (AV, ≤3 mg KOH/g), POV (≤5 meq/kg), p-AV (≤20), and TOTOX (≤26) throughout the stated lifetime of a product [15]. We found that the VA and SuF tested in this study met their stated label claims for GOED (Table 3). POV and AV are used in the industry’s on-line quality control index. POV has a significant correlation to the off-flavour compounds created during the initial oxidation. AV represents the content of free fatty acids which are easily oxidized to hydro-peroxides [28].

The EC regulations suggest that absorbances at 234 nm (K234), 270 nm (K270), and 280 nm (K280) be used to measure oils according to their oxidated products such as ethylenic diketones, conjugated ketodienes, and the dienal formation of conjugated dienes and trienes. K234 is a primary oxidation index that has been found to be closely related to hydroperoxide content [29]. In addition, the measurement of UV absorbance at 270 and 280 nm has been used previously in analyzing edible oils. Absorption at these wavelengths is mainly due to secondary oxidation. In a spectral analysis conducted for this study (Table 3), we found that the absorbances of 10 mg/mL of VA, SuF, and SiF were 3.33, 14.79, and 7.58, respectively, at K234; 1.12, 0.66, and 1.78, respectively, at K270; and 1.08, 0.51 and 1.48, respectively, at K280. Most of them increased as the oxidation levels increased. The K234 of SuF had high values at the beginning, a finding which can be attributed to the residual oxidated products or fat-soluble compounds that were produced from the simplified refining process.

**Figure 3:**
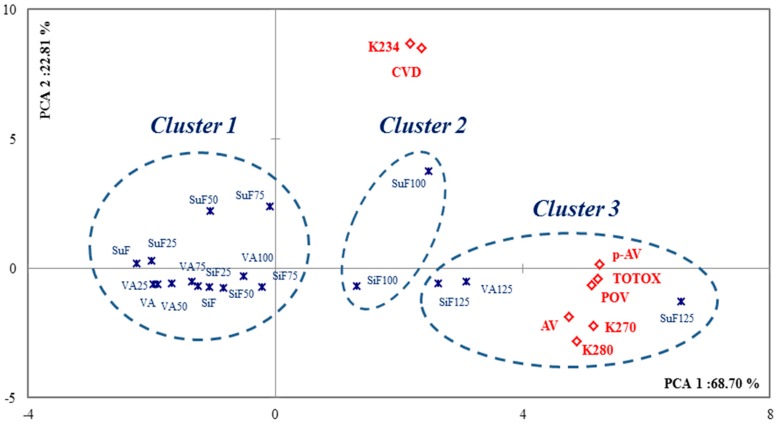
PCA plots of quality changes for the different oxidation levels of the *n*-3 PUFA-rich oils under the Rancimat method; the circles represent the clusters detected with AHC analysis; the solid fill type highlights the values with PCA.

In the literatures, hierarchical cluster was showed that could apply to grouping basis on quality, sensory attributes and refined level [30,31]. The AHC analysis was applied to identify clusters of samples with similar oxidation properties. Three main clusters were extracted (Figure 3). Cluster 1 contained the 0–100% oxidation levels of the VA, the 0–75% oxidation levels of the SuF, and the 0–75% oxidation levels of the SiF. Cluster 2 contained the 100% oxidation levels of the SuF and SiF. Cluster 3 contained the 125% oxidation levels of the VA, SuF, and SiF. These results showed that there are significant changes of quality in the IP of a given oil when it is subjected to the Rancimat test. These results were similar to those of a previous study reported by our team, which oxidized volatiles of *n*-3 PUFA [32].

With regard to the quality standard change at 80 °C under the Rancimat test, according to the PCA analysis, two dimensions were extracted and together account for approximately 91.51% of the variability from the original data (Figure 3). In our results, we observed p-AV (r: 0.92), TOTOX (r: 0.91), K270 (r: 0.88), and POV (r: 0.87) with PAC1; and K234 (r: 0.83) and conjugated dienes (CVD, r: 0.81) with PCA2. The POV and p-AV are measures of primary oxidation and secondary oxidation, respectively. The TOTOX value gives an overall indication of the complete oxidation status of oil, and consists of the combination of the POV and the p-AV values to determine the oxidation level of the oil. Correlations between the quality parameters and spectral analysis is that K234, with AV, POV, p-AV, and TOTOX is low (*R* < 0.5), while K270 and K280 with acid value (AV), POV, p-AV, and TOTOX is high (*R* > 0.7). These results were based on measuring the formation of secondary oxidation products; for example, p-AV and K270 are available to support the AV evaluation of frying oil quality, and aldehyde molecules were a common marker for K270, K280, and p-AV [28,33].

## 3. Materials and Methods

### 3.1. Materials

Conventional algae oils (VA) were provided by VEDAN Enterprise Corporation (Taichung, Taiwan), and conventional fish oils from mackerel (SuF) were provided by SUN AGRICULTURE (Ilan, Taiwan). Additional fish oils (SiF) were provided by Sigma (Sigma-Aldrich, Taufkirchen, Germany). A fatty acid methyl ester standard (FAME) mixture, Supelco 37 Component FAME Mix, was purchased from Supelco (Sigma-Aldrich, Taufkirchen, Germany). Standards of α-, γ-, and δ- tocopherol were purchased from Merck (Darmstadt, Germany).

### 3.2. Rancimat Test

The oxidative stability index of the *n*-3 PUFA-rich oil was previously determined at four different temperatures (70 °C, 80 °C, 90 °C, and 100 °C) as the induction period (hours) that was recorded using a Rancimat 743 apparatus and a 5 ± 0.05 g sample of oil with an air flow of 10 L/h. Then, oil samples were oxidized at 80 °C for periods of time that corresponded to 25%, 50%, 75%, 100%, and 125% of their respective induction periods. The oil samples that were used to determine the oxidative stability were also analyzed for their volatile oxidation compounds. The IP of the *n*-3 PUFA-rich oils were automatically recorded and taken as the break point of the plotted curves (the intersection point of the two extrapolated parts of the curve).

### 3.3. Kinetic Data Analysis

The kinetic parameters were determined according to the method previously utilized as reported in [17]. The IP of the oil samples were automatically recorded and taken as the break point of the plotted curves (the intersection point of the two extrapolated parts of the curve). A kinetic rate constant was taken as the inverse of the IP (*k*, h^−1^).

Temperature coefficients (*T* Coeff, K^−1^) were determined from the slopes of the lines generated by regressing ln(*k*) vs. the absolute temperature (*T*, K):

ln(*k*) = *a*(*T*) + *b* (1)

where *a* and *b* are the equation parameters.

Activation energies (*E*a, kJ/mol) and pre-exponential or frequency factors (*A*, h^−1^) were determined from the slopes and intercepts, respectively, of the lines generated by regressing ln(*k*) vs. 1/*T* using the Arrhenius equation:

ln(*k*) = ln(*A*) − (*E*a/R*T*) (2)

where *k* is the reaction rate constant or reciprocal IP (h^−1^), and R is the molar gas constant (8.3143 J/mol K).

### 3.4. Analysis of Tocopherol

Each oil sample (0.1 g) was diluted with 2-propanol to a volume of 10 mL and filtered through an MS nylon syringe filter with a 0.45 μm pore size directly to vials and then immediately analyzed using an HPLC system. Aliquots of 10 µL of the filtrate were injected into the injection port and analyzed with HPLC (Hitachi L-2130 pump, Hitachi, Tokyo, Japan). The remaining procedures were carried out as previously reported [34] using an HPLC attached to a detector L-2400 UV and a Hitachi L-2130 pump. An RP-18GP250 Mightysil column (*l* = 250 mm; i.d. = 4.6 mm; thickness = 0.32 µm; Kanto Chemical Co., Inc., Tokyo, Japan) was used for separation. The same mobile phase and elution conditions were adopted. The calibration curves were, respectively, established for tocopherol by plotting the peak area vs. each corresponding concentration, from which quantitations of the standards were achieved.

### 3.5. Fatty Acid Analysis

The *n*-3 PUFA-rich oil was analyzed for its fatty acid composition via GC/FID. The triacylglycerols were converted to methyl esters using the AOCS Official Method Ce 2–66 [35]. The methyl esters were separated using a column that was coated with DB-23 (30 m × 0.25 mm × 0.25 μm, Agilent, Palo Alto, CA, USA), and helium was used as the carrier gas at a flow rate of 1.0 mL/min. The oven temperature was initially held for 8 min at 200 °C, and then increased at 10 °C/min to 220 °C and then held there for 40 min. The FID was maintained at 270 °C, and the injector (split mode 1:40, 4 mm liner) was maintained at 250 °C. The contents of the fatty acids were determined using the normalization method, with heneicosanoic methyl ester used as an internal standard to quantitation.

### 3.6. Quality Analytical Determination

The POV and UV spectrophotometric were measured using the analytical methods described in European Regulation EEC 2568/91 [36]. The CDA measures the formed from PUFA during lipid oxidation according to the AOCS method Ti la-64 [35]. The p-AV measures secondary oxidation products, such as 2-alkenal and 2,4-alkadienal. The p-AV of each sample was determined according to the AOCS method Cd 18–90 [35]. The AV was determined using a titration with 0.1 N potassium hydroxide alcoholic solution.

### 3.7. Statistical Analysis

The data reported were obtained from triplicate measurements of each sample and were expressed as means. The data were subjected to an AHC analysis with squared Euclidean distances. Subsequently, the data were analyzed using PCA combined with VARIMAX rotation. For the AHC and PCA analysis, XLSTAT software (version 2010.2.01, Addinsoft Deutschland, Andernach, Germany) was used.

## 4. Conclusions

In this work, the Rancimat test was applied to the analysis of the oxidation properties of *n*-3 PUFA-rich oil. The results of the present study indicated: (1) the degree of unsaturation in fatty acids is not the only parameter for assessing oil quality, which is mainly influenced by commercialization. The oxidation stability of *n*-3 PUFA-rich oils is improved by commercialization, especially with respect to antioxidant protection; and (2) chemometric applications have also clarified the nature of the differences in correlation among oxidative and chemical parameters. The IP of primary and secondary oxidation product formations represented the quality changes as detected via AHC analysis. Not all quality parameters increased linearly through PCA analysis, which can allow for the selection of a proper and adequate method for a particular application. It is recommended for p-AV, TOTOX, K270, and POV to be used when carrying out quality control for *n*-3 PUFA- rich oils.

## Figures and Tables

**Table 1:** Regression parameters for Arrhenius relationships between the reaction rate constant and the temperature for the *n*-3 PUFA-rich oils.

Groups	VA	SuF	SiF
ln(*k*) = *a*(1/*T*) + *b*			
*a*	−11.66	−11.66	−9.96
*b*	31.16	31.1	27.44
*R*^2^	0.973	0.971	0.998
*E*a (kJ/mol)	96.98	96.97	82.84

**Table 2:** The fatty acid composition and tocopherol levels of the *n*-3 PUFA-rich oils.

Groups	VA	SuF	SiF
**Fatty Acid (g/100 g)**			
**SFA ^a^**	**34.4**	**44.5**	**43.9**
C14:0	2.6	8.6	15.6
C16:0	30.2	29.8	25.6
C18:0	1.6	6.1	2.7
**MUFA ^b^**	**34.5**	**20.2**	**24.0**
C14:1	1.3	1.9	1.1
C16:1	N.D ^d^	4.8	18.0
C18:1	33.2	11.7	7.1
C20:1	N.D	1.8	1.8
**PUFA ^c^**	**30.3**	**17.6**	**24.0**
C18:2	3.5	1.2	3.5
C20:2	N.D	N.D	3.3
C20:3	N.D	1.9	0.6
AA	N.D	N.D	0.9
EPA	N.D	4.6	9.9
DPA	3.5	1.0	1.1
DHA	23.3	8.9	4.7
**Tocopherol (mg/kg)**			
δ-	219.3	252.9	167.3
γ-	529.4	445.3	N.D
α-	106.6	124.4	N.D

^a^ SFA, Saturated fatty acid; ^b^ MUFA, monounsaturated fatty acid; ^c^ PUFA, polyunsaturated fatty acid; ^d^ N.D, not detected.

**Table 3:** Initial quality characteristics of the *n*-3 PUFA-rich oils.

Groups	VA	SuF	SiF
**Quality Indicators**			
AV (mg KOH/g)	0.48 ± 0.02	0.49 ± 0.01	0.65 ± 0.01
CVD (%)	0.22 ± 0.05	1.18 ± 0.17	0.58 ± 0.03
POV (meq/kg)	1.98 ± 0.27	4.12 ± 0.34	13.62 ± 0.42
p-AV (meq/kg)	6.33 ± 0.71	15.12 ± 0.64	29.23 ± 1.84
TOTOX (meq/kg)	10.30 ± 0.92	23.26 ± 1.24	56.46 ± 2.11
**Visible Spectra (10 mg/mL)**			
K234 ^a^	3.33 ± 0.07	14.79 ± 0.14	7.58 ± 0.17
K270	1.12 ± 0.01	0.66 ± 0.02	1.78 ± 0.04
K280	1.08 ± 0.02	0.51 ± 0.01	1.48 ± 0.07

^a^ K234, K270, and K280, specific absorption at 234, 270, and 280 nm.

## Abbreviations

The following abbreviations are used in this manuscript:

SFA	Saturated fatty acids
MUFA	Monounsaturated fatty acid
PUFA	Polyunsaturated fatty acid
AA	Arachidonic acid
EPA	Eicosapentaenoic acid
DPA	Docosapentaenoic acid
DHA	Docosahexaenoic acid
GOED	Global Organization for EPA and DHA
IP	Induction period
*E*a	Activation energies
POV	Peroxide value
p-AV	Anisidine value
AV	Acid value
CD	Conjugated dienes
TOTOX	Total oxidation value
PCA	Principal component analysis
AHC	Agglomerative hierarchical cluster
